# Catalyzing marketing innovation and competitive advantage in the healthcare industry: the value of thinking like an outsider

**DOI:** 10.1186/s12913-018-3682-9

**Published:** 2018-12-14

**Authors:** James K. Elrod, John L. Fortenberry

**Affiliations:** 1 Willis-Knighton Health System, 2600 Greenwood Road, Shreveport, LA 71103 USA; 2 LSU Shreveport, 1 University Place, Shreveport, LA 71115 USA

**Keywords:** Marketing, Innovation, Competitive advantage, Hospitals, Healthcare

## Abstract

**Background:**

Marketing arguably is the most critical administrative responsibility associated with the pursuit and realization of growth and prosperity, making prowess in the discipline essential for any healthcare institution, especially given the competitive intensity that characterizes the industry. But in order to truly gain an advantage, healthcare establishments must tap into innovative pathways that their competitors have yet to discover. Here, thinking like an outsider can pay tremendous dividends, as health and medical organizations tend to focus inwardly, limiting their exposure to externally-derived innovations and advancements which often can supply differentiation opportunities.

**Discussion:**

Some years ago, during a formative period in preparation for expanding its footprint, Willis-Knighton Health System opted to think like an outsider, peering beyond the walls of healthcare institutions in search of tools and techniques that would allow its growth ambitions to be realized. Associated pursuits and subsequent successes created a culture of challenging status quo perspectives, affording innovations and resulting competitive advantages. Marketing advancements, in particular, have been fueled by this outsider mentality, benefiting the institution and its patient populations. This article profiles several of these advancements, discusses the dangers of insular mindsets, and suggests avenues for encouraging broad perspectives.

**Conclusions:**

Due to extreme competitive intensity and ever-increasing patient needs, health and medical establishments must perform at optimal levels, with marketing efforts playing a critical role in the achievement of such. By shedding status quo perspectives and peering beyond the walls of healthcare institutions, health and medical providers have opportunities to discover new and different marketing approaches for potential use in their own organizations, affording mutual benefits, including all-important competitive advantages.

## Background

Formally defined, marketing is “a management process that involves the assessment of customer wants and needs, and the performance of all activities associated with the development, pricing, provision, and promotion of product solutions that satisfy those wants and needs” [1], p. 288. Close examination of this definition reveals that the discipline is both wide and deep. Specifically, the definition (1) notes that marketing is a process, meaning that it is ongoing and must actively be managed; (2) brings attention to the Four Ps—Product, Price, Place, Promotion—which must be formulated for each target audience; (3) indicates that the focus is on the consumer; and (4) conveys that products—goods and services—are used to satisfy customer wants and needs, implying product development and management, and the necessity to effect exchange. Marketing arguably is the most critical administrative responsibility associated with the pursuit and realization of growth and prosperity, making prowess in the discipline essential for any healthcare institution, especially given the competitive intensity that characterizes the industry [1, 2].

But in order to truly gain an advantage, establishments must tap into innovative pathways that their competitors have yet to discover [1, 3–6]. Here, thinking like an outsider can pay tremendous dividends, as health and medical organizations tend to focus inwardly, limiting their exposure to externally-derived innovations and advancements which often can supply differentiation opportunities [7]. Outside-the-box thinking also seems to be in short supply often times, presenting yet another opportunity to achieve distinction. Such insular mindsets should not be particularly surprising to astute observers of the healthcare industry, as health and medical personnel typically work hand-in-hand with others engaged in like pursuits, hold memberships in healthcare-related professional societies, subscribe to newsletters and other publications which focus on health and medicine, and attend conferences focused on healthcare topics, limiting their exposure to innovations and advancements originating in other industries and fostering mindsets centered squarely on developments within their given work environments [7–12]. But in this very characteristic of the healthcare industry lies opportunity for those enterprising health and medical establishments which dare to think like outsiders [7, 13].

## Discussion

Beginning in the 1970s, during a formative period in preparation for expanding its footprint, Willis-Knighton Health System opted to think like an outsider, peering beyond the walls of healthcare institutions in search of tools and techniques that would allow its growth ambitions to be realized. Outside-the-box thinking also was encouraged, unleashing intensive creativity which afforded groundbreaking innovations, producing windfall benefits. Among other things, the institution turned to various structure, product, and process innovations, adopting the hub-and-spoke model of organization design [14, 15], establishing centers of excellence [16], and embracing the practice of adaptive reuse [17, 18], with each of these approaches notably emerging from outside of the healthcare industry [7]. Successes experienced on these fronts were complemented by a range of equivalent successes in marketing, with each of these being derived not from following common pathways which looked within the healthcare industry for solutions, but by pushing the envelope of creative thought and action, assuming the role of outsider in search of novel advancements permitting extensive competitive advantages. Notable examples of such pursuits are as follows.

- ***Pioneering health services advertising***: In the 1970s, Willis-Knighton Health System deployed advertising years in advance of the healthcare industry’s full acceptance and use of the medium. Advertising was viewed during this period as being beneath the dignity of medical organizations, with some also frowning on the practice due to its potential to upset the traditional method of patient acquisition: referrals between and among caregivers [1, 3, 6, 19]. Noting advertising’s widespread deployment by virtually all other industries, Willis-Knighton Health System forged a new and different pathway, affording competitive advantages which fueled growth while status quo market participants lost ground.
- ***Modeling patient experiences after hotel guest experiences***: Desiring customer service excellence, Willis-Knighton Health System turned to the hotel industry for insights permitting enhanced patient experiences, noting parallels between hospital patients and hotel guests (e.g., both are away from home, both are immersed in unfamiliar environments). This led to the provision of a number of value-added offerings, including concierge services, complimentary lodging, and free transportation, greatly elevating customer service, attention, and support, with the spark igniting this innovative array of services being an industry far removed from delivering health and medical care [13].
- ***Taking a road less traveled to bolster target marketing efforts***: In 1979, Willis-Knighton Health System identified and pursued an off the beaten path in its bid to capture market share in pediatric healthcare services. The direct route—targeting current and prospective parents—was heavily pursued by competitors, prompting the institution to seek a road less traveled which would reach the same audiences but do so via a different route. Children, as direct care recipients, supplied one such route, prompting the institution to develop an associated bond. Painstaking efforts yielded Willis-Knighton Health System’s Pediatric Orientation Program, fostering an affinity between the institution and children, which in turn influenced parents, affording opportunities for enhanced patronage in pediatric medicine and beyond [13].
- ***Expanding the boundaries of traditional branding thought***: To complement existing branding initiatives, Willis-Knighton Health System sought identity opportunities outside the boundaries of traditional branding thought, leading it to develop a branded stuffed animal—Willis the Bear—to promote labor and delivery services [13]. The institution’s iconic teddy bear mascot made his official debut on Mother’s Day 2001. Presented exclusively to mothers who deliver their babies at Willis-Knighton Health System, Willis the Bear significantly elevated awareness of the institution’s labor and delivery services, illustrating the power and utility of supplementing traditional branding pursuits with nontraditional, expanded perspectives.
- ***Becoming an owner-operator of digital billboards***: In 2018, Willis-Knighton Health System initiated an innovative communications pursuit, effectively entering the outdoor advertising business by installing digital billboards at several of its locations. This endeavor paired institutional assets (e.g., excellent locations, outstanding roadside visibility, high traffic counts) with a relatively new technology (i.e., digital billboards) to create a unique marketing communications asset. Unlike the fairly common digital signage fronting many establishments, Willis-Knighton Health System replicated the size and appearance of the digital billboards used by outdoor advertising companies, presenting a familiar format to passersby and achieving a high communications impact, yielding numerous competitive advantages.
- ***Encouraging personnel to view themselves from the perspective of patients***: Willis-Knighton Health System has encouraged patient attentiveness and empathy throughout its history, investing heavily in assets designed to facilitate the best patient experiences possible [13]. Given the vital role of health and medical personnel in achieving exceptional patient care and support, the institution suggests a reflective exercise that encourages staff members to see themselves and their actions from the perspective of patients. Asking the operative question, “Am I seeing things through the eyes of patients?” serves as an effective reminder of priorities, building empathy and motivating personnel to continually deliver their very best. In many respects, viewing oneself from the perspective of patients represents the ultimate example of thinking like an outsider.

The marketing innovations and resulting competitive advantages afforded by the pursuits and practices noted above would never have been realized without shedding internal industry mindsets in favor of more global perspectives. Profoundly impacting the state of innovation at Willis-Knighton Health System, this broad, inquisitive view fostered new ways of addressing challenges which amplified performance and ultimately supplied numerous mutual benefits. An external focus, effectively representing a window to the outside world, is imperative for examining the state of innovation across business and industry, nurturing ideas that might potentially be transferred for use within healthcare establishments and encouraging creativity that could possibly yield fruitful discoveries and resulting applications [7, 13].

As for achieving an outsider mentality, creative thinking indeed is essential [13, 20]. This, of course, must be complemented by a receptiveness to new and different ideas and a willingness to experiment. Assuming one has the potential for creative thought and is situated in a healthcare organization welcoming of innovative ideas and associated experimentation, the final ingredient is exposure to broader perspectives. This can be achieved by engaging in environmental scanning, making conscious efforts to look beyond the healthcare industry for innovations and advancements emerging in other venues, all the while considering how observed ideas might be used within health and medical institutions [7, 13].

This does not require significant alterations in one’s daily work life. Reading trade publications which address audiences from across business and industry, expanding personal and professional networks to include those serving in industries other than healthcare, immersing oneself in greater society to observe developments, and similar engagements will afford significant exposure to perspectives far and wide. For added benefits, information sharing sessions can be conducted within healthcare establishments, permitting staff members to discuss observations gleaned from their broad searches and ponder potential application opportunities in their given organizations. These and related efforts have greatly facilitated Willis-Knighton Health System’s marketing performance, demonstrating the power of thinking like an outsider as a means of fostering marketing innovation and competitive advantage.

## Conclusions

Due to extreme competitive intensity and ever-increasing patient needs, health and medical establishments must perform at optimal levels, with marketing efforts playing a critical role in the achievement of such. Insular mindsets which direct attention solely toward the healthcare industry are harmful, as they restrict much needed exposure to the full range of advancements occurring in broad business and industry. By shedding status quo perspectives and peering beyond the walls of healthcare institutions, health and medical providers have opportunities to discover new and different marketing approaches for potential use in their own organizations. As Willis-Knighton Health System discovered, catalyzing marketing innovation and competitive advantage indeed is possible by thinking like an outsider.
